# Undocumented Migrants Reintroducing COVID-19, Yunnan Province, China

**DOI:** 10.3201/eid2705.204944

**Published:** 2021-05

**Authors:** Meiling Zhang, Jienan Zhou, Senquan Jia, Xiaonan Zhao, Yaoyao Chen, Yanhong Sun, Zhaosheng Liu, Xiaofang Zhou, Duo Li, Chunrui Luo, Yong Zhang, Violet Magoma Onsongo, Yong Shao, Xiaoqing Fu

**Affiliations:** Yunnan Provincial Center for Disease Control and Prevention, Kunming, China (M.L. Zhang, J.N. Zhou, S.Q. Jia, X.N. Zhao, Y.Y. Chen, Y.H. Sun, Z.S. Liu, X.F. Zhou, D. Li, C.R. Luo, Y. Zhang, X.Q. Fu);; Kunming Institute of Zoology, Kunming (V.M. Onsongo, Y. Shao)

**Keywords:** SARS-CoV-2, migrants, China, COVID-19, respiratory infections, severe acute respiratory syndrome coronavirus 2, 2019 novel coronavirus disease, coronavirus disease, zoonoses, viruses, coronaviruses, Yunnan Province, borders, migration

## Abstract

To limit the spread of severe acute respiratory syndrome coronavirus 2, the government of China has been monitoring infected travelers and minimizing cold-chain contamination. However, other factors might contribute to recurring outbreaks. We analyze the role of undocumented migrants as potential transmitters of severe acute respiratory syndrome coronavirus 2 in China.

China’s efforts to suppress coronavirus disease (COVID-19), the illness caused by severe acute respiratory syndrome coronavirus 2 (SARS-CoV-2), rely on rigorous quarantine measures. These measures contributed to a decline in COVID-19 cases; no new locally acquired cases were reported in China on March 18, 2020 (http://www.nhc.gov.cn/xcs/yqtb/list_gzbd_10.shtml). As a result, the focus of epidemic control and prevention work has shifted from local to imported cases of COVID-19. Although viral spread has been contained by mandates minimizing travel and cold-chain contamination (*1*), recurring COVID-19 outbreaks might be caused by other factors and pathways. On September 14, 2020, the discovery of 2 SARS-CoV-2–infected undocumented migrants from Myanmar prompted large-scale testing of >280,000 persons in Ruili, Yunnan Province, China (Figure).

**Figure:**
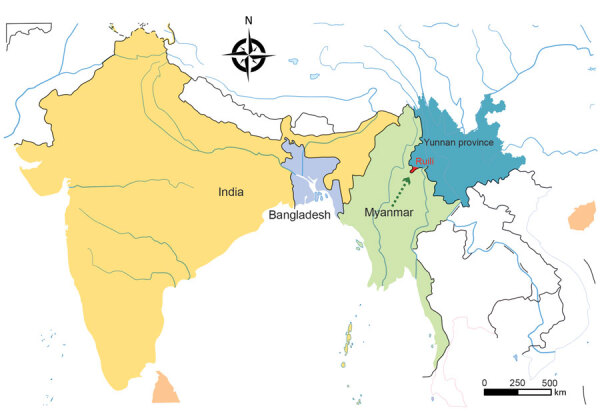
Ruili, Yunnan Province, China, in relation to neighboring countries. Map from http://bzdt.ch.mnr.gov.cn. Arrow indicates direction of migration.

On March 31, 2020, the Yunnan Provincial Leading Group for COVID-19 Epidemic Response published Notice No. 15 (http://www.yn.gov.cn/ztgg/yqfk/zcfk/202004/t20200401_201604.html), which outlined strict measures to prevent COVID-19 importation from land and water ports. This notice discouraged citizens of adjacent countries from entering Yunnan Province; if entry was required, then those citizens should enter Yunnan Province via 1 of 19 official land ports (Appendix Table 1). Before continuing their travels, these persons needed to undergo 14 days of quarantine and test negative for SARS-CoV-2 by nucleic acid amplification test. On September 3, 2020, two undocumented migrants with no history of rejection at any official port crossed the Ruili River from Nankan (Myanmar) to Ruili. 

Patient 1, who had lost her senses of smell and taste for 1 week before diagnosis, received a COVID-19 diagnosis on September 12, 2020, ending a 139-day period in which no new cases had been reported in Yunnan Province. In Myanmar, she had not had contact with any known COVID-19 patient. Officials identified 201 close contacts of patient 1 in Yunnan Province; these contacts were then tested for SARS-CoV-2. All contacts tested negative except the person who had entered China with patient 1. Because of the 9-day delay between entry and diagnosis, whether community transmission had occurred was unknown. 

To evaluate potential spread, we undertook a large-scale SARS-CoV-2 screening campaign of >280,000 citizens and legal migrants in Ruili during September 15–19, 2020. We did not detect any cases of community transmission, possibly because of patient 1’s low viral load; she had a mild case of COVID-19, with normal lung physiology and an N-gene cycle threshold of 37.41, suggesting a low level of infectiousness (*2*) (Appendix Figure 1). Patient 1 also wore a mask in public, potentially hindering COVID-19 transmission. In addition, patient 2 did not spend much time in public, further reducing potential for transmission. Although this event did not cause community spread of SARS-CoV-2 in Ruili, it highlights the need to curb undocumented immigration to prevent recurring outbreaks of COVID-19. This need is especially relevant in Yunnan Province, which shares a 4,060-km border with Myanmar, Laos, and Vietnam. The border spans 8 cities and 25 counties of China.

To evaluate potential variations in SARS-CoV-2 sequences for the 2 cases, we conducted whole-genome sequencing (*3*) of high-quality reads mapped to a reference sequence from Wuhan (GenBank accession no. MN908947.3). We deposited consensus sequences in GISAID (https://www.gisaid.org) under accession nos. EPI_ISL_632934 and EPI_ISL_632935. Sequence alignment analyses (*4*) revealed that the 2 SARS-CoV-2 sequences from Ruili shared 13 mutations: C241T, C3037T, G11083T, C14408T, G18756T, C18877T, C22444T, A23403G, G25494T, G25563T, C26735T, C28854T, and G29737C (Appendix Figure 2) (*5*,*6*). According to the Pangolin COVID-19 Lineage Assigner (*7*), 9 of these mutations (i.e., C241T, C3037T, C14408T, C18877T, C22444T, A23403G, G25563T, C26735T, and C28854T) indicate membership in the B.1.36 clade of SARS-CoV-2. Further phylogenetic analyses supported this conclusion (Appendix Table 2, Figure 3). Compared with sequences from earlier COVID-19 outbreaks in Beijing Xinfadi Market (*1*,*8*), Dalian (*9*), and Qingdao, the Ruili sequences had 7 previously unreported mutations (Appendix Figure 2, panel B) (*5*). The Ruili cases were not associated with the mentioned outbreaks and were probably imported.

Although the SARS-CoV-2–infected migrants did not cause a COVID-19 outbreak, the event illustrates a transmission pathway distinct from air travel and cold-chain food transmission (*1*). The International Health Regulations and World Health Organization encourage open borders and suggest that COVID-19 control measures be applied only in limited circumstances (*10*). In 2020, official land ports in Yunnan Province did not close for the COVID-19 pandemic. Because of the long international border, epidemic control remains challenging in this province. Governments should control illegal immigration to avoid future reintroductions of COVID-19. Regional guidelines for COVID-19 control and prevention should strengthen surveillance of undocumented movement across borders, especially from neighboring countries with high rates of infection.

Appendix Additional information on undocumented migrants reintroducing COVID-19.
