# Supplementary material for: Undocumented Migrants Reintroducing COVID-19, Yunnan Province, China
Source: Emerg Infect Dis. 2021 May;27(5):1543–5. doi: 10.3201/eid2705.204944 (PMC8084479; doi:10.3201/eid2705.204944)
Supplement: Appendix — Additional information on undocumented migrants reintroducing COVID-19. [file 20-4944-Techapp-s1.pdf]

Article DOI: https://doi.org/10.3201/eid2705.204944

Undocumented Migrants Reintroducing COVID-19, Yunnan Province, China

Appendix

Activity Log of Patient 1

On September 3, 2020, patient 1 arrived at the Aoxingshiji community after sneaking into Ruili, Yunnan Province, China and remained indoors that night. On September 4, she shopped at the mall and returned home. On September 5, patient 1 stayed at home. On September 6, she went to the mall in the evening, visited Nongmohu Park, and returned home. On September 7, patient 1 visited relatives, went to a Taibeichangdi restaurant, and returned home. On September 8, patient 1 stayed at home. On September 9, patient 1 went to the gym to exercise and then returned home. On September 10, she sought care at the Department of Cardiology, Ruili People's Hospital (Ruili, China) for heart discomfort, and then went to the outpatient department of Jingcheng Hospital in Ruili, where she fraudulently used another person's identifying information. She provided a sample for nucleic acid amplification test and returned home. On September 11, patient 1 stayed at home. On September 12, she received the positive result of the reverse transcription PCR (RT-PCR) for severe acute respiratory syndrome coronavirus 2 (SARS-CoV-2) from Jingcheng Hospital and was then transferred to Ruili People's Hospital by a negative pressure ambulance for treatment and isolation.

Activity Log of Patient 2

Patient 2 was the servant of patient 1. She arrived at the Aoxingshiji community after sneaking into Ruili with patient 1 on September 3, 2020 and remained indoors that night. On

September 4–5, patient 2 stayed at home. On September 6, patient 2 went to Nongmohu Park with patient 1 and returned home. On September 7–11, patient 2 stayed at home. On September 12, when technicians were conducting nucleic acid amplification tests on close contacts of patient 1, patient 2's result of SARS-CoV-2 was found to be positive. Patient 2 was transferred to Ruili People's Hospital by a negative pressure ambulance for treatment and isolation.

SARS-CoV-2 RNA Detection

We extracted total nucleic acids using an automatic preparation system (NP968, Tianlong, http://www.medtl.com). We analyzed the nucleic acids by real-time quantitative RT-PCR according to the protocol recommended by China National Health Commission (Appendix Figure 1) (*1*).

Sequencing Library Construction

We conducted 1-step amplification for the nucleic acids using the SARS-COV-2 genome-wide capture amplification kit (V-090418, MicroFuture, http://www.vmfuture.com).We purified the PCR amplification products with the MinElute PCR Purification Kit (QIAGEN, https://www.qiagen.com) and qualified their concentrations with the Invitrogen Qubit 2.0 Fluorometer (Thermo Fisher Scientific, https://www.thermofisher.com). Then, we constructed the complementary DNA libraries using Nextera XT DNA Library Preparation Kit (Illumina, Inc., https://www.illumina.com) according to the manufacturer's instructions and sequenced them on the Illumina MiSeq platform with 2 × 150 paired-end mode using 300-cycle MiSeq version 2 reagent kits (Illumina, Inc.).

Sequencing Data Analysis

We sequenced >4.5 Gb; >90% had a base quality score >Q30, indicating a <0.1% error rate. We trimmed the raw sequencing reads using CLC Genomics Workbench version 12

software (QIAGEN) with quality scores ≥0.05 and ambiguous nucleotides ≤2. We trimmed the 5′ sequencing adaptor by removing 5 terminal nucleotides and the 3′ by removing 18 terminal nucleotides. Then, we mapped the high-quality reads to the Wuhan-Hu-1 reference (GenBank accession no. MN908947.3) using the following mapping options: match score = 1; mismatch cost = 2 (linear gap cost); length fraction = 0.5; similarity fraction = 0.8; autodetect paired distance; nonspecific match handling: map randomly. We obtained the consensus sequences for downstream analysis.

Evolutionary and Mutational Analysis

We downloaded 110,596 high-quality reference sequences from 6 continents from the GISAID database (https://www.gisaid.org) on September 15, 2020. To ensure that analyses were robust, we randomly extracted 1 sequence per month (collection date) from each country. Including the Wuhan-Hu-1 reference and 2 Ruili sequences, a total of 592 sequences were aligned by using MAFFT version 7 (*2*) using the default options (Appendix Table 2). We obtained the mutation sites using the robust alignments. Then we applied RAxML version 8 (*3*) to construct the evolutionary phylogeny using the GTRGAMMA model under a bootstrap value of 200.

References

<eref>1. Chinese Center for Disease Control and Prevention. Guidelines of laboratory testing for novel coronavirus in 2019. 2020 [cited 2020 Aug 16]. http://www.chinacdc.cn/jkzt/crb/zl/szkb_11803/jszl_11815/202003/t20200309_214241. html </eref>

<jrn>2. Katoh K, Misawa K, Kuma K, Miyata T. MAFFT: a novel method for rapid multiple sequence alignment based on fast Fourier transform. Nucleic Acids Res. 2002;30:3059–66. PubMed https://doi.org/10.1093/nar/gkf436</jrn>

<jrn>3. Stamatakis A. RAxML version 8: a tool for phylogenetic analysis and post-analysis of large phylogenies. Bioinformatics. 2014;30:1312–3. PubMed https://doi.org/10.1093/bioinformatics/btu033</jrn>

Appendix Table 1. Official land ports, Yunnan Province, China, 2020

No.	Name
1	Houqiao of Baoshan
2	Hekou highway of Honghe
3	Hekou railway of Honghe
4	Jinshuihe of Honghe
5	Tianbao of Wenshan
6	Dulong of Wenshan
7	Tianpeng of Wenshan
8	Menga of Puer
9	Mengkang of Puer
10	Mohan of Xishuangbanna
11	Daluo of Xishuangbanna
12	Ruili of Dehong
13	Wanding of Dehong
14	Zhangfeng of Dehong
15	Nabang of Dehong
16	Pianma of Nujiang
17	Qingshuihe of Lincang
18	Yonghe of Lincang
19	Nansan of Lincang

Appendix Table 2. Comparison of severe acute respiratory syndrome coronavirus 2 sequences from various sources and undocumented migrants, Yunnan Province, China, 2020

GISAID ID	Strain	Collection date*
EPI_ISL_417186	hCoV-19/South Africa/R03006/2020	2020 Mar 7
EPI_ISL_417941	hCoV-19/DRC/73/2020	2020 Mar 18
EPI_ISL_431011	hCoV-19/DRC/1319/2020	2020 Apr 7
EPI_ISL_455412	hCoV-19/Nigeria/OG007-CV22/2020	2020 Mar 29
EPI_ISL_455431	hCoV-19/Nigeria/OY045A-CV35/2020	2020 Apr 2
EPI_ISL_463001	hCoV-19/Tunisia/COV0010-12/2020	2020 Mar 18
EPI_ISL_463004	hCoV-19/Tunisia/COV1663/2020	2020 Apr 1
EPI_ISL_467433	hCoV-19/South Africa/KRISP-0017/2020	2020 Apr 3
EPI_ISL_483035	hCoV-19/Egypt/MASRI-009/2020	2020 Apr 30
EPI_ISL_483036	hCoV-19/Egypt/MASRI-018/2020	2020 May 10
EPI_ISL_485635	hCoV-19/Senegal/68349/2020	2020 Jun 18
EPI_ISL_487446	hCoV-19/Mali/M002593/2020	2020 Apr 6
EPI_ISL_498100	hCoV-19/South Africa/KRISP-0561/2020	2020 Jul 15
EPI_ISL_529800	hCoV-19/South Africa/KRISP-K002806/2020	2020 Aug 25
EPI_ISL_561134	hCoV-19/Gambia/GC19-2707/2020	2020 Jul 21
EPI_ISL_451183	hCoV-19/Uganda/UG001/2020	2020 Mar 25
EPI_ISL_451400	hCoV-19/Morocco/OUA677-19/2020	2020 Apr 23
EPI_ISL_467500	hCoV-19/South Africa/KRISP-0150/2020	2020 May 30
EPI_ISL_467501	hCoV-19/South Africa/KRISP-0151/2020	2020 Jun 1
EPI_ISL_471456	hCoV-19/Morocco/HMIMV-Rabat102-03/2020	2020 Mar 31
EPI_ISL_477161	hCoV-19/Egypt/CUNCI-HGC6I029/2020	2020 Jun 2
EPI_ISL_480783	hCoV-19/Senegal/1683/2020	2020 Mar 31
EPI_ISL_480789	hCoV-19/Senegal/1966/2020	2020 Apr 1
EPI_ISL_527901	hCoV-19/Nigeria/OY605-CV170/2020	2020 Jun 12
EPI_ISL_451197	hCoV-19/Uganda/UG015/2020	2020 Apr 27
EPI_ISL_471158	hCoV-19/Gambia/0214/2020	2020 Mar 29
EPI_ISL_471163	hCoV-19/Gambia/0536/2020	2020 Apr 30
EPI_ISL_471167	hCoV-19/Gambia/1094/2020	2020 May 4
EPI_ISL_476822	hCoV-19/Benin/197/2020	2020 Mar 15
EPI_ISL_476825	hCoV-19/Benin/1408/2020	2020 Apr 4
EPI_ISL_487101	hCoV-19/Nigeria/OY260-CV55/2020	2020 May 16
EPI_ISL_496492	hCoV-19/DRC/5240/2020	2020 May 3
EPI_ISL_498238	hCoV-19/Senegal/45436/2020	2020 May 27
EPI_ISL_515100	hCoV-19/Ghana/82879_S48/2020	2020 May 20
EPI_ISL_524426	hCoV-19/Egypt/CUNCI-7I028/2020	2020 Jul 19
EPI_ISL_561038	hCoV-19/Gambia/GC19-1983/2020	2020 Jun 7

GISAID ID	Strain	Collection date*
EPI_ISL_568847	hCoV-19/Kenya/C7605/2020	2020 May 16
EPI_ISL_568872	hCoV-19/Kenya/C21582/2020	2020 Jun 22
EPI_ISL_581486	hCoV-19/Congo/UKT-001/2020	2020 Apr
EPI_ISL_581487	hCoV-19/Congo/UKT-002/2020	2020 Jul 4
EPI_ISL_581488	hCoV-19/Congo/UKT-004/2020	2020 May
EPI_ISL_581489	hCoV-19/Congo/UKT-005/2020	2020 Jun 6
EPI_ISL_602800	hCoV-19/South Africa/KRISP-K003466/2020	2020 Sep 13
EPI_ISL_457827	hCoV-19/Kenya/NIC_059/2020	2020 Mar
EPI_ISL_458150	hCoV-19/Morocco/15N/2020	2020 May 15
EPI_ISL_508862	hCoV-19/Madagascar/IP-01650/2020	2020 Mar 20
EPI_ISL_515181	hCoV-19/Ghana/KATH23/2020	2020 Mar 22
EPI_ISL_539573	hCoV-19/Gabon/ITM-K011/2020	2020 Mar 22
EPI_ISL_418241	hCoV-19/Algeria/G0638_2264/2020	2020 Mar 2
EPI_ISL_451202	hCoV-19/Uganda/UG020/2020	2020 May 1
EPI_ISL_457854	hCoV-19/Kenya/C1216/2020	2020 Apr 19
EPI_ISL_476559	hCoV-19/Morocco/refstage1/2020	2020 Feb 27
EPI_ISL_510529	hCoV-19/Zambia/29/2020	2020 Mar 16
EPI_ISL_522547	hCoV-19/Reunion/RUN-PIMIT1/2020	2020 Mar 24
EPI_ISL_526975	hCoV-19/Egypt/EGY-020/2020	2020 Aug 14
EPI_ISL_527873	hCoV-19/Nigeria/ED01-CV155/2020	2020 Feb 21
EPI_ISL_418206	hCoV-19/Senegal/003/2020	2020 Feb 28
EPI_ISL_512811	hCoV-19/Sierra Leone/KGH-G-8548/2020	2020 Mar 30
EPI_ISL_512813	hCoV-19/Sierra Leone/KGH-G-8603/2020	2020 Apr 23
EPI_ISL_561288	hCoV-19/Gambia/NPHL-2712/2020	2020 Aug 3
EPI_ISL_605780	hCoV-19/Egypt/C-CEIRS-19 MOH/2020	2020 Mar 13
EPI_ISL_560386	hCoV-19/Botswana/BOT0455/2020	2020 Mar 28
EPI_ISL_591086	hCoV-19/DRC/16191/2020	2020 Jun 3
EPI_ISL_539496	hCoV-19/Andorra/202552/2020	2020 Mar 13
EPI_ISL_437932	hCoV-19/Austria/CeMM0045/2020	2020 Feb 24
EPI_ISL_437996	hCoV-19/Austria/CeMM0148/2020	2020 Mar 2
EPI_ISL_475824	hCoV-19/Austria/CeMM0381/2020	2020 Apr 1
EPI_ISL_583847	hCoV-19/Austria/CeMM1007/2020	2020 May 2
EPI_ISL_583883	hCoV-19/Austria/CeMM1077/2020	2020 Jun 30
EPI_ISL_583885	hCoV-19/Austria/CeMM1083/2020	2020 Jul 19
EPI_ISL_583887	hCoV-19/Austria/CeMM1091/2020	2020 Aug 3
EPI_ISL_419692	hCoV-19/Belarus/ChVir2072/2020	2020 Mar
EPI_ISL_407976	hCoV-19/Belgium/GHB-03021/2020	2020 Feb 3
EPI_ISL_418987	hCoV-19/Belgium/CG-030158/2020	2020 Mar 1

GISAID ID	Strain	Collection date*
EPI_ISL_434371	hCoV-19/Belgium/ITM_C233/2020	2020 Apr 1
EPI_ISL_476996	hCoV-19/Belgium/rega-0501480/2020	2020 May 1
EPI_ISL_475082	hCoV-19/Belgium/UGent-122/2020	2020 Jun 1
EPI_ISL_498149	hCoV-19/Belgium/ULG-10243/2020	2020 Jul 3
EPI_ISL_540507	hCoV-19/Belgium/ULG-10340/2020	2020 Aug 4
EPI_ISL_582129	hCoV-19/Belgium/ITM-C5184/2020	2020 Sep 13
EPI_ISL_462450	hCoV-19/Bosnia and Herzegovina/ChVir7340/2020	2020 Mar 17
EPI_ISL_462472	hCoV-19/Bosnia and Herzegovina/ChVir7382/2020	2020 Apr 1
EPI_ISL_462753	hCoV-19/Bosnia and Herzegovina/01-Livno/2020	2020 May 27
EPI_ISL_467778	hCoV-19/Bucuresti/ChVir7244/2020	2020 Apr 27
EPI_ISL_480310	hCoV-19/Bulgaria/41/2020	2020 Mar 10
EPI_ISL_480300	hCoV-19/Bulgaria/17/2020	2020 Apr 14
EPI_ISL_480302	hCoV-19/Bulgaria/21/2020	2020 May 7
EPI_ISL_428901	hCoV-19/Crimea/SRC-80603/2020	2020 Mar 23
EPI_ISL_429659	hCoV-19/Croatia/OY-S1/2020	2020 Mar
EPI_ISL_454574	hCoV-19/Croatia/OY-S1new/2020	2020 Apr 9
EPI_ISL_463741	hCoV-19/Cyprus/001/2020	2020 Mar 11
EPI_ISL_463745	hCoV-19/Cyprus/005/2020	2020 Apr 1
EPI_ISL_416742	hCoV-19/Czech Republic/ChVir1630/2020	2020 Feb 29
EPI_ISL_414477	hCoV-19/Czech Republic/951/2020	2020 Mar 1
EPI_ISL_491118	hCoV-19/Czech Republic/NRL_5752/2020	2020 Apr 1
EPI_ISL_584073	hCoV-19/Czech Republic/NRL-6847-2/2020	2020 May 10
EPI_ISL_541082	hCoV-19/Czech Republic/NRL_7000/2020	2020 Jun 10
EPI_ISL_546436	hCoV-19/Czech Republic/NRL-7950/2020	2020 Jul 22
EPI_ISL_546935	hCoV-19/Czech Republic/NRL-8118-1/2020	2020 Aug 5
EPI_ISL_584081	hCoV-19/Czech Republic/NRL-8598/2020	2020 Sep 3
EPI_ISL_416142	hCoV-19/Denmark/SSI-01/2020	2020 Feb 26
EPI_ISL_416144	hCoV-19/Denmark/SSI-03/2020	2020 Mar 1
EPI_ISL_437630	hCoV-19/Denmark/ALAB-SSI-1046/2020	2020 Apr 1
EPI_ISL_452098	hCoV-19/Denmark/ALAB-HH-267/2020	2020 May 9
EPI_ISL_407071	hCoV-19/England/01/2020	2020 Jan 29
EPI_ISL_464302	hCoV-19/England/201040021/2020	2020 Feb 3
EPI_ISL_417213	hCoV-19/England/20102068502/2020	2020 Mar 1
EPI_ISL_432727	hCoV-19/England/SHEF-C1645/2020	2020 Apr 1
EPI_ISL_448391	hCoV-19/England/NOTT-110B74/2020	2020 May 1
EPI_ISL_472057	hCoV-19/England/LIVE-9FCE4/2020	2020 Jun 1
EPI_ISL_493608	hCoV-19/England/NOTT-112CCA/2020	2020 Jul 1
EPI_ISL_514346	hCoV-19/England/203290063/2020	2020 Aug 1

GISAID ID	Strain	Collection date*
EPI_ISL_540753	hCoV-19/England/SHEF-CB03E/2020	2020 Sep 1
EPI_ISL_420540	hCoV-19/Estonia/ChVir2148/2020	2020 Mar
EPI_ISL_526937	hCoV-19/Faroe Islands/HFS-04/2020	2020 Mar 12
EPI_ISL_407079	hCoV-19/Finland/1/2020	2020 Jan 29
EPI_ISL_413602	hCoV-19/Finland/FIN03032020A/2020	2020 Mar 3
EPI_ISL_481649	hCoV-19/Finland/2A92SVIIC/2020	2020 Apr 2
EPI_ISL_481674	hCoV-19/Finland/4May21S4/2020	2020 May 4
EPI_ISL_406596	hCoV-19/France/IDF-0372/2020	2020 Jan 23
EPI_ISL_411218	hCoV-19/France/IDF-0571/2020	2020 Feb 2
EPI_ISL_434616	hCoV-19/France/OCC-1/2020	2020 Mar
EPI_ISL_443279	hCoV-19/France/BRE-5434/2020	2020 Apr 1
EPI_ISL_510527	hCoV-19/France/PAC-FR/2020	2020 May 1
EPI_ISL_568994	hCoV-19/France/PAC-IHU-0928/2020	2020 Jun 5
EPI_ISL_568998	hCoV-19/France/PAC-IHU-0942/2020	2020 Jul 2
EPI_ISL_560607	hCoV-19/France/BRE-8939/2020	2020 Aug 1
EPI_ISL_603218	hCoV-19/France/OCC-82/2020	2020 Sep 30
EPI_ISL_450201	hCoV-19/Germany/BY-ChVir-1247/2020	2020 Jan
EPI_ISL_450203	hCoV-19/Germany/BY-ChVir-1289/2020	2020 Feb 1
EPI_ISL_602518	hCoV-19/Germany/NW-HHU-148/2020	2020 Mar 1
EPI_ISL_437278	hCoV-19/Germany/BY-MVP-0084/2020	2020 Apr 1
EPI_ISL_466905	hCoV-19/Germany/BY-MVP-0251/2020	2020 May 2
EPI_ISL_487410	hCoV-19/Germany/NW-MPP-21/2020	2020 Jun 5
EPI_ISL_539596	hCoV-19/Germany/NW-HHU-80/2020	2020 Aug 16
EPI_ISL_547444	hCoV-19/Gibraltar/203360729/2020	2020 Jul 21
EPI_ISL_547436	hCoV-19/Gibraltar/203260482/2020	2020 Aug 3
EPI_ISL_430469	hCoV-19/Greece/127_HPI/2020	2020 Feb 29
EPI_ISL_437894	hCoV-19/Greece/234_31670/2020	2020 Mar 5
EPI_ISL_437910	hCoV-19/Greece/52_37266/2020	2020 Apr 1
EPI_ISL_501233	hCoV-19/Greece/19553_HPI/2020	2020 May 3
EPI_ISL_418183	hCoV-19/Hungary/mbl2/2020	2020 Mar 17
EPI_ISL_435418	hCoV-19/Hungary/SRC-01136/2020	2020 Apr 2
EPI_ISL_526236	hCoV-19/Hungary/MH-6603/2020	2020 May 8
EPI_ISL_526224	hCoV-19/Hungary/MH-9653/2020	2020 Jun 4
EPI_ISL_526225	hCoV-19/Hungary/MH-13211/2020	2020 Jul 6
EPI_ISL_417765	hCoV-19/Iceland/13/2020	2020 Feb 27
EPI_ISL_424367	hCoV-19/Iceland/343/2020	2020 Mar
EPI_ISL_414586	hCoV-19/Ireland/LK-NVRL-19934/2020	2020 Mar 3
EPI_ISL_437684	hCoV-19/Ireland/un-NVRL-20W266/2020	2020 Apr

GISAID ID	Strain	Collection date*
EPI_ISL_501259	hCoV-19/Ireland/LH-NVRL-20IRL22956/2020	2020 May 27
EPI_ISL_500573	hCoV-19/Ireland/SO-NVRL-70IRL90620/2020	2020 Jul 2
EPI_ISL_525371	hCoV-19/Ireland/CN-NVRL-71IRL96106/2020	2020 Aug 1
EPI_ISL_578308	hCoV-19/Ireland/KK-NVRL-73IRL21709/2020	2020 Sep 4
EPI_ISL_410545	hCoV-19/Italy/LAZ-INMI1-isl/2020	2020 Jan 29
EPI_ISL_451300	hCoV-19/Italy/LAZ-INMI1-N/2020	2020 Feb 3
EPI_ISL_417418	hCoV-19/Italy/FVG-ICGEB-S1/2020	2020 Mar 1
EPI_ISL_452189	hCoV-19/Italy/VEN-IZSVe-33-126/2020	2020 Apr 1
EPI_ISL_522859	hCoV-19/Italy/VEN-IZSVe-51635/2020	2020 May 1
EPI_ISL_525572	hCoV-19/Italy/APU-UniMI-64PT/2020	2020 Jun 1
EPI_ISL_498558	hCoV-19/Italy/FVG-ICGEB-S208/2020	2020 Jul
EPI_ISL_584072	hCoV-19/Italy/CAM-IZSM-183/2020	2020 Sep 12
EPI_ISL_419691	hCoV-19/Latvia/ChVir2025/2020	2020 Mar
EPI_ISL_450519	hCoV-19/Latvia/019/2020	2020 Apr 14
EPI_ISL_486391	hCoV-19/Latvia/026/2020	2020 May 3
EPI_ISL_501286	hCoV-19/Latvia/070/2020	2020 Jun 5
EPI_ISL_492998	hCoV-19/Latvia/063/2020	2020 Jul 3
EPI_ISL_515196	hCoV-19/Latvia/109/2020	2020 Aug 3
EPI_ISL_416741	hCoV-19/Lithuania/ChVir1632/2020	2020 Feb
EPI_ISL_450496	hCoV-19/Lithuania/ChVir2225/2020	2020 Mar
EPI_ISL_541868	hCoV-19/Lithuania/MR-LUHS-Eilnr6/2020	2020 Apr 2
EPI_ISL_560401	hCoV-19/Lithuania/C20-05-R5/2020	2020 May 18
EPI_ISL_560405	hCoV-19/Lithuania/C20-06-R20/2020	2020 Jun 3
EPI_ISL_413593	hCoV-19/Luxembourg/Lux1/2020	2020 Feb 29
EPI_ISL_419566	hCoV-19/Luxembourg/LNS0641910/2020	2020 Mar 5
EPI_ISL_429722	hCoV-19/Luxembourg/LNS6137379/2020	2020 Apr 1
EPI_ISL_445075	hCoV-19/Luxembourg/LNS1586475/2020	2020 May 8
EPI_ISL_576117	hCoV-19/Malta/BAL-Sliema-1/2020	2020 Aug 19
EPI_ISL_576118	hCoV-19/Malta/BAL-Sliema-2/2020	2020 Sep 4
EPI_ISL_523968	hCoV-19/Moldova/ChVir7776/2020	2020 Mar 11
EPI_ISL_516938	hCoV-19/Moldova/ICGEB_MD6/2020	2020 Jun 17
EPI_ISL_516922	hCoV-19/Montenegro/ChVir-1265622002/2020	2020 Mar 17
EPI_ISL_516925	hCoV-19/Montenegro/ChVir-1793232002/2020	2020 Jul 17
EPI_ISL_454750	hCoV-19/Netherlands/NoordHolland_10001/2020	2020 Feb 27
EPI_ISL_413572	hCoV-19/Netherlands/Haarlem_1363688/2020	2020 Mar 1
EPI_ISL_422600	hCoV-19/Netherlands/NA_291/2020	2020 Apr 1
EPI_ISL_460835	hCoV-19/Netherlands/Flevoland_12/2020	2020 May 1
EPI_ISL_523134	hCoV-19/Netherlands/FL-EMC-25/2020	2020 Jun 1

GISAID ID	Strain	Collection date*
EPI_ISL_523659	hCoV-19/Netherlands/ZH-EMC-317/2020	2020 Jul 1
EPI_ISL_523712	hCoV-19/Netherlands/ZH-EMC-372/2020	2020 Aug 1
EPI_ISL_577944	hCoV-19/Netherlands/ZH-EMC-516/2020	2020 Sep 1
EPI_ISL_514354	hCoV-19/North Macedonia/2175/2020	2020 Jun 2
EPI_ISL_516428	hCoV-19/North Macedonia/6497/2020	2020 Jul 8
EPI_ISL_441410	hCoV-19/Northern Ireland/NIRE-1023D7/2020	2020 Mar 10
EPI_ISL_585175	hCoV-19/Northern Ireland/NIRE-10822F/2020	2020 Apr 1
EPI_ISL_469848	hCoV-19/Northern Ireland/NIRE-100EE7/2020	2020 May 1
EPI_ISL_488876	hCoV-19/Northern Ireland/NIRE-FF0B9/2020	2020 Jun 1
EPI_ISL_532578	hCoV-19/Northern Ireland/QEUH-94390D/2020	2020 Jul 11
EPI_ISL_532561	hCoV-19/Northern Ireland/QEUH-943493/2020	2020 Aug 1
EPI_ISL_585226	hCoV-19/Northern Ireland/NIRE-FB30F/2020	2020 Sep 1
EPI_ISL_590667	hCoV-19/Northern Ireland/QEUH-9B8192/2020	2020 Sep 3
EPI_ISL_417484	hCoV-19/Norway/1380/2020	2020 Feb 26
EPI_ISL_417486	hCoV-19/Norway/1538/2020	2020 Mar 1
EPI_ISL_449793	hCoV-19/Norway/2388/2020	2020 Apr 2
EPI_ISL_471176	hCoV-19/Norway/2534/2020	2020 May 4
EPI_ISL_549173	hCoV-19/Norway/3171/2020	2020 Jun 19
EPI_ISL_500773	hCoV-19/Norway/2850/2020	2020 Jul 2
EPI_ISL_549059	hCoV-19/Norway/2957/2020	2020 Aug 1
EPI_ISL_590903	hCoV-19/Norway/3280/2020	2020 Sep 2
EPI_ISL_435723	hCoV-19/Poland/Wro-01/2020	2020 Mar
EPI_ISL_455441	hCoV-19/Poland/PL_P28/2020	2020 Apr 1
EPI_ISL_451644	hCoV-19/Poland/Pom3/2020	2020 May 2
EPI_ISL_485399	hCoV-19/Poland/IHG_PAS_4_67/2020	2020 Jun 1
EPI_ISL_582031	hCoV-19/Poland/IHG-PAS-8-29/2020	2020 Sep 5
EPI_ISL_413647	hCoV-19/Portugal/CV62/2020	2020 Mar 1
EPI_ISL_454211	hCoV-19/Portugal/PT0487/2020	2020 Apr 1
EPI_ISL_454221	hCoV-19/Portugal/PT0497/2020	2020 May 1
EPI_ISL_491217	hCoV-19/Portugal/IGC3996/2020	2020 Jun 1
EPI_ISL_468134	hCoV-19/Romania/Bucuresti-4105/2020	2020 Mar 21
EPI_ISL_491085	hCoV-19/Romania/ROSV_569/2020	2020 Apr 5
EPI_ISL_491086	hCoV-19/Romania/ROSV_5812/2020	2020 May 1
EPI_ISL_471416	hCoV-19/Romania/Buzau-291946/2020	2020 Jun 1
EPI_ISL_467780	hCoV-19/Romania /ChVir7246/2020	2020 Apr 26
EPI_ISL_428860	hCoV-19/Russia/Moscow-62505/2020	2020 Mar 11
EPI_ISL_428878	hCoV-19/Russia/Omsk-89001/2020	2020 Apr 1
EPI_ISL_507205	hCoV-19/Russia/Bryansk-RII24784S/2020	2020 May

GISAID ID	Strain	Collection date*
EPI_ISL_524022	hCoV-19/Russia/SPE-RII-18966V/2020	2020 Jun 1
EPI_ISL_569828	hCoV-19/Russia/OMS-ORINFI-2112S/2020	2020 Jul 2
EPI_ISL_596353	hCoV-19/Russia/PRI-RII-MH733S/2020	2020 Aug 6
EPI_ISL_596233	hCoV-19/Russia/LEN-RII-34268M/2020	2020 Sep
EPI_ISL_413221	hCoV-19/Scotland/CVR01/2020	2020 Feb 28
EPI_ISL_425807	hCoV-19/Scotland/CVR77/2020	2020 Mar 3
EPI_ISL_433200	hCoV-19/Scotland/EDB573/2020	2020 Apr 1
EPI_ISL_439337	hCoV-19/Scotland/EDB3846/2020	2020 May 1
EPI_ISL_478154	hCoV-19/Scotland/CVR3828/2020	2020 Jun 1
EPI_ISL_490704	hCoV-19/Scotland/CVR3940/2020	2020 Jul 3
EPI_ISL_514537	hCoV-19/Scotland/EDB7118/2020	2020 Aug 1
EPI_ISL_540786	hCoV-19/Scotland/CVR3989/2020	2020 Sep 1
EPI_ISL_462434	hCoV-19/Serbia/NS838-04/2020	2020 Apr 1
EPI_ISL_462435	hCoV-19/Serbia/KV22-05/2020	2020 May 10
EPI_ISL_541656	hCoV-19/Serbia/KV-0140707/2020	2020 Jul 6
EPI_ISL_516988	hCoV-19/Slovakia/ChVir-1996/2020	2020 Mar
EPI_ISL_572329	hCoV-19/Slovakia/UKBA-101/2020	2020 Apr 3
EPI_ISL_577739	hCoV-19/Slovakia/UKBA-208/2020	2020 May 6
EPI_ISL_577740	hCoV-19/Slovakia/UKBA-209/2020	2020 Jun 30
EPI_ISL_577734	hCoV-19/Slovakia/UKBA-201/2020	2020 Jul 9
EPI_ISL_583481	hCoV-19/Slovakia/UKBA-313/2020	2020 Sep 10
EPI_ISL_420541	hCoV-19/Slovenia/808/2020	2020 Mar 5
EPI_ISL_449799	hCoV-19/Slovenia/MB0419/2020	2020 Apr 19
EPI_ISL_539531	hCoV-19/Spain/CN-ISCIII-201048/2020	2020 Feb 24
EPI_ISL_418245	hCoV-19/Spain/CM-ISCIII-201328/2020	2020 Mar 1
EPI_ISL_452371	hCoV-19/Spain/AN-IBV-002058/2020	2020 Apr 1
EPI_ISL_510448	hCoV-19/Spain/AN-IBV-006119/2020	2020 May 2
EPI_ISL_481100	hCoV-19/Spain/MD-IBV-004983/2020	2020 Jun 1
EPI_ISL_510439	hCoV-19/Spain/AN-IBV-006108/2020	2020 Jul 2
EPI_ISL_541904	hCoV-19/Spain/MD-IBV-99007131/2020	2020 Aug 1
EPI_ISL_582054	hCoV-19/Spain/PV-IBV-98007584/2020	2020 Sep1
EPI_ISL_476139	hCoV-19/Sweden/20-02114/2020	2020 Jan 31
EPI_ISL_411951	hCoV-19/Sweden/01/2020	2020 Feb 7
EPI_ISL_455848	hCoV-19/Sweden/20-50056/2020	2020 Mar 2
EPI_ISL_434657	hCoV-19/Sweden/20-06813/2020	2020 Apr 1
EPI_ISL_469055	hCoV-19/Sweden/20-14262/2020	2020 May 2
EPI_ISL_475568	hCoV-19/Sweden/20-51816/2020	2020 Jun 1
EPI_ISL_534235	hCoV-19/Sweden/20-52288/2020	2020 Jul 1

GISAID ID	Strain	Collection date*
EPI_ISL_534232	hCoV-19/Sweden/20-08950/2020	2020 Aug 2
EPI_ISL_615115	hCoV-19/Sweden/20-52829/2020	2020 Sep 4
EPI_ISL_413996	hCoV-19/Switzerland/TI-SNRCI-29919486/2020	2020 Feb 24
EPI_ISL_414023	hCoV-19/Switzerland/VD-SNRCI-29965615/2020	2020 Mar 1
EPI_ISL_486442	hCoV-19/Switzerland/BS-ETHZ-110000/2020	2020 Apr 1
EPI_ISL_476105	hCoV-19/Switzerland/BE-ETHZ-140055/2020	2020 May 1
EPI_ISL_581907	hCoV-19/Switzerland/BL-42292327/2020	2020 Jun 1
EPI_ISL_489973	hCoV-19/Switzerland/ZH-ETHZ-180031/2020	2020 Jul 1
EPI_ISL_581933	hCoV-19/Switzerland/BS-42376604/2020	2020 Aug 1
EPI_ISL_541513	hCoV-19/Switzerland/ZH-ETHZ-270047/2020	2020 Sep 1
EPI_ISL_429866	hCoV-19/Turkey/HSGM-4236/2020	2020 Mar 16
EPI_ISL_478670	hCoV-19/Turkey/KOC-IST-B91/2020	2020 Apr 8
EPI_ISL_480253	hCoV-19/Turkey/GLAB-CoV155/2020	2020 May 1
EPI_ISL_495436	hCoV-19/Turkey/KU-026/2020	2020 Jun 3
EPI_ISL_512640	hCoV-19/Ukraine/203100361/2020	2020 Apr 24
EPI_ISL_512636	hCoV-19/Ukraine/203100357/2020	2020 May 8
EPI_ISL_512597	hCoV-19/Ukraine/203100317/2020	2020 Jun 23
EPI_ISL_512616	hCoV-19/Ukraine/203100337/2020	2020 Jul 10
EPI_ISL_576148	hCoV-19/Ukraine/Kyiv-785/2020	2020 Aug 3
EPI_ISL_513310	hCoV-19/United Kingdom/USAFSAM-S026/2020	2020 Mar 18
EPI_ISL_413555	hCoV-19/Wales/PHW1/2020	2020 Feb 27
EPI_ISL_432308	hCoV-19/Wales/PHWC-24E39/2020	2020 Mar
EPI_ISL_431944	hCoV-19/Wales/PHWC-28EF9/2020	2020 Apr 1
EPI_ISL_474675	hCoV-19/Wales/PHWC-364E7/2020	2020 May 1
EPI_ISL_473239	hCoV-19/Wales/PHWC-163064/2020	2020 Jun 1
EPI_ISL_557846	hCoV-19/Wales/ALDP-6AE860/2020	2020 Jul 1
EPI_ISL_514577	hCoV-19/Wales/PHWC-169ACA/2020	2020 Aug 1
EPI_ISL_540899	hCoV-19/Wales/PHWC-16A15B/2020	2020 Sep 1
EPI_ISL_402123	hCoV-19/Wuhan/IPBCAMS-WH-01/2019	2019 Dec 24
EPI_ISL_413691	hCoV-19/Weifang/WF0001/2020	2020 Jan
EPI_ISL_403928	hCoV-19/Wuhan/IPBCAMS-WH-05/2020	2020 Jan 1
EPI_ISL_411929	hCoV-19/South Korea/SNU01/2020	2020 Jan
EPI_ISL_434560	hCoV-19/Hong Kong/HK20/2020	2020 Jan
EPI_ISL_414511	hCoV-19/Japan/TKYE6182/2020	2020 Jan
EPI_ISL_514752	hCoV-19/Beijing/Beijing-01/2020	2020 Jan 3
EPI_ISL_437623	hCoV-19/Thailand/SI200040-NT/2020	2020 Jan 8
EPI_ISL_412459	hCoV-19/Jingzhou/HBCDC-HB-01/2020	2020 Jan 8
EPI_ISL_406030	hCoV-19/Shenzhen/HKU-SZ-002/2020	2020 Jan 10

GISAID ID	Strain	Collection date*
EPI_ISL_421252	hCoV-19/Pingxiang/JX5/2020	2020 Jan 11
EPI_ISL_408486	hCoV-19/Jiangxi/IVDC-JX-002/2020	2020 Jan 11
EPI_ISL_410301	hCoV-19/Nepal/61/2020	2020 Jan 13
EPI_ISL_403932	hCoV-19/Guangdong/20SF012/2020	2020 Jan 14
EPI_ISL_408484	hCoV-19/Sichuan/IVDC-SC-001/2020	2020 Jan 15
EPI_ISL_404227	hCoV-19/Zhejiang/WZ-01/2020	2020 Jan 16
EPI_ISL_539333	hCoV-19/Hunan/HN-CIDC-P2/2020	2020 Jan 17
EPI_ISL_408480	hCoV-19/Yunnan/IVDC-YN-003/2020	2020 Jan 17
EPI_ISL_408481	hCoV-19/Chongqing/IVDC-CQ-001/2020	2020 Jan 18
EPI_ISL_407313	hCoV-19/Hangzhou/HZCDC0001/2020	2020 Jan 19
EPI_ISL_408482	hCoV-19/Shandong/IVDC-SD-001/2020	2020 Jan 19
EPI_ISL_408488	hCoV-19/Jiangsu/IVDC-JS-001/2020	2020 Jan 19
EPI_ISL_411060	hCoV-19/Fujian/8/2020	2020 Jan 21
EPI_ISL_416389	hCoV-19/Shanghai/SH0093/2020	2020 Jan 21
EPI_ISL_421243	hCoV-19/Nanchang/JX14/2020	2020 Jan 21
EPI_ISL_406533	hCoV-19/Guangzhou/20SF206/2020	2020 Jan 22
EPI_ISL_406534	hCoV-19/Foshan/20SF207/2020	2020 Jan 22
EPI_ISL_418267	hCoV-19/Vietnam/19-02S/2020	2020 Jan 22
EPI_ISL_421237	hCoV-19/Jiujiang/JX22/2020	2020 Jan 22
EPI_ISL_421244	hCoV-19/Shangrao/JX29/2020	2020 Jan 22
EPI_ISL_406973	hCoV-19/Singapore/1/2020	2020 Jan 23
EPI_ISL_406031	hCoV-19/Taiwan/2/2020	2020 Jan 23
EPI_ISL_495459	hCoV-19/Lishui/LS003/2020	2020 Jan 24
EPI_ISL_416866	hCoV-19/Malaysia/MKAK-CL-2020-5047/2020	2020 Jan 24
EPI_ISL_421242	hCoV-19/Ganzhou/JX81/2020	2020 Jan 25
EPI_ISL_421249	hCoV-19/Xinyu/JX122/2020	2020 Jan 25
EPI_ISL_455680	hCoV-19/Changzhou/JS27/2020	2020 Jan 26
EPI_ISL_421253	hCoV-19/Jian/JX129/2020	2020 Jan 26
EPI_ISL_463889	hCoV-19/Shaoxing/01/2020	2020 Jan 27
EPI_ISL_413522	hCoV-19/India/MH-1-27/2020	2020 Jan 27
EPI_ISL_411902	hCoV-19/Cambodia/0012/2020	2020 Jan 27
EPI_ISL_582125	hCoV-19/United Arab Emirates/skmc-920168117/2020	2020 Jan 28
EPI_ISL_429104	hCoV-19/Guangzhou/GZMU0036/2020	2020 Feb 1
EPI_ISL_455466	hCoV-19/Yingtan/JX2480/2020	2020 Feb 1
EPI_ISL_468724	hCoV-19/Japan/TKYE6938/2020	2020 Feb
EPI_ISL_413711	hCoV-19/Weifang/WF0014/2020	2020 Feb
EPI_ISL_507039	hCoV-19/South Korea/CBNU-nCoV01/2020	2020 Feb
EPI_ISL_437624	hCoV-19/Thailand/SI200893-NT/2020	2020 Feb 1

GISAID ID	Strain	Collection date*
EPI_ISL_412030	hCoV-19/Hong Kong/VB20026565-2/2020	2020 Feb 1
EPI_ISL_416336	hCoV-19/Shanghai/SH0027/2020	2020 Feb 1
EPI_ISL_407988	hCoV-19/Singapore/3/2020	2020 Feb 1
EPI_ISL_413861	hCoV-19/Guangdong/GD2020080-P0010/2020	2020 Feb 1
EPI_ISL_452360	hCoV-19/Beijing/DT-WH01/2020	2020 Feb 1
EPI_ISL_429854	hCoV-19/Lishui/LS557/2020	2020 Feb 1
EPI_ISL_451316	hCoV-19/Sichuan/SC-GA-065/2020	2020 Feb 1
EPI_ISL_416047	hCoV-19/Hangzhou/ZJU-06/2020	2020 Feb 2
EPI_ISL_421257	hCoV-19/Shangrao/JX1215/2020	2020 Feb 2
EPI_ISL_455463	hCoV-19/Jiujiang/JX490/2020	2020 Feb 3
EPI_ISL_454919	hCoV-19/Wuhan/HB-WH1-143/2020	2020 Feb 3
EPI_ISL_489996	hCoV-19/Saudi Arabia/477/2020	2020 Feb 3
EPI_ISL_416886	hCoV-19/Malaysia/MKAK-CL-2020-6430/2020	2020 Feb 4
EPI_ISL_455462	hCoV-19/Fuzhou/JX2012/2020	2020 Feb 5
EPI_ISL_463901	hCoV-19/Shaoxing/09/2020	2020 Feb 5
EPI_ISL_410218	hCoV-19/Taiwan/NTU02/2020	2020 Feb 5
EPI_ISL_455461	hCoV-19/Yichun/JX2391/2020	2020 Feb 6
EPI_ISL_435134	hCoV-19/United Arab Emirates/L4280/2020	2020 Feb 8
EPI_ISL_412983	hCoV-19/Tianmen/HBCDC-HB-07/2020	2020 Feb 8
EPI_ISL_416429	hCoV-19/Vietnam/CM99/2020	2020 Feb 11
EPI_ISL_412026	hCoV-19/Hefei/2/2020	2020 Feb 23
EPI_ISL_457701	hCoV-19/Oman/RESP-20-797/2020	2020 Feb 23
EPI_ISL_419211	hCoV-19/Israel/ISR_JP0320/2020	2020 Feb 23
EPI_ISL_450442	hCoV-19/Henan/HN03/2020	2020 Feb 24
EPI_ISL_421246	hCoV-19/Nanchang/JXN3T4/2020	2020 Feb 26
EPI_ISL_450512	hCoV-19/Lebanon/S5_762/2020	2020 Feb 27
EPI_ISL_415641	hCoV-19/Georgia/Tb-54/2020	2020 Feb 27
EPI_ISL_468725	hCoV-19/Japan/TKYE63442/2020	2020 Mar
EPI_ISL_437604	hCoV-19/Thailand/SI206377-NST/2020	2020 Mar 1
EPI_ISL_447330	hCoV-19/Israel/51137031/2020	2020 Mar 1
EPI_ISL_418999	hCoV-19/Singapore/18/2020	2020 Mar 1
EPI_ISL_490012	hCoV-19/Saudi Arabia/677/2020	2020 Mar 1
EPI_ISL_454918	hCoV-19/Wuhan/HB-WH1-142/2020	2020 Mar 1
EPI_ISL_497997	hCoV-19/South Korea/KCDC2096/2020	2020 Mar 1
EPI_ISL_431101	hCoV-19/India/TG-GMC-RK100/2020	2020 Mar 1
EPI_ISL_596539	hCoV-19/Palestine/60/2020	2020 Mar
EPI_ISL_413592	hCoV-19/Taiwan/NTU03/2020	2020 Mar 2
EPI_ISL_459909	hCoV-19/Harbin/HRB-26/2020	2020 Mar 2

GISAID ID	Strain	Collection date*
EPI_ISL_416458	hCoV-19/Kuwait/KU12/2020	2020 Mar 2
EPI_ISL_457706	hCoV-19/Oman/RESP-20-1189/2020	2020 Mar 2
EPI_ISL_596453	hCoV-19/Iran/GRC-43/2020	2020 Mar 2
EPI_ISL_450508	hCoV-19/Lebanon/S1_758/2020	2020 Mar 4
EPI_ISL_417444	hCoV-19/Pakistan/Gilgit1/2020	2020 Mar 4
EPI_ISL_501181	hCoV-19/Malaysia/0956/2020	2020 Mar 4
EPI_ISL_452351	hCoV-19/Beijing/DT-travelIT04/2020	2020 Mar 4
EPI_ISL_416479	hCoV-19/Georgia/Tb-273/2020	2020 Mar 5
EPI_ISL_482575	hCoV-19/Hangzhou/HZCDC6111/2020	2020 Mar 5
EPI_ISL_429239	hCoV-19/Yunnan/0306-466/2020	2020 Mar 6
EPI_ISL_497791	hCoV-19/Hong Kong/HKU-200723-024/2020	2020 Mar 6
EPI_ISL_416430	hCoV-19/Vietnam/CM295/2020	2020 Mar 6
EPI_ISL_483547	hCoV-19/Bahrein/BAH-06/2020	2020 Mar 7
EPI_ISL_424352	hCoV-19/Fuyang/FY002/2020	2020 Mar 10
EPI_ISL_435129	hCoV-19/United Arab Emirates/L1758/2020	2020 Mar 10
EPI_ISL_428671	hCoV-19/Sri Lanka/COV53/2020	2020 Mar 10
EPI_ISL_443187	hCoV-19/Brunei/1/2020	2020 Mar 11
EPI_ISL_529961	hCoV-19/Indonesia/JI-ITD-136N/2020	2020 Mar 12
EPI_ISL_431118	hCoV-19/Fujian/IM3520001T/2020	2020 Mar 13
EPI_ISL_430002	hCoV-19/Jordan/SR-044/2020	2020 Mar 16
EPI_ISL_435045	hCoV-19/Kazakhstan/NCB-1/2020	2020 Mar 22
EPI_ISL_417420	hCoV-19/NanChang/JX216/2020	2020 Mar 23
EPI_ISL_427408	hCoV-19/Qatar/QA13/2020	2020 Mar 23
EPI_ISL_455688	hCoV-19/Zhejiang/OS1/2020	2020 Mar 24
EPI_ISL_434555	hCoV-19/Philippines/PGC03/2020	2020 Mar 26
EPI_ISL_456600	hCoV-19/Timor-Leste/TL25/2020	2020 Mar 30
EPI_ISL_513000	hCoV-19/Saudi Arabia/KAUST-JEDDAH757/2020	2020 Apr 1
EPI_ISL_485396	hCoV-19/South Korea/S5/2020	2020 Apr 1
EPI_ISL_507026	hCoV-19/Saudi Arabia/KAIMRC62/2020	2020 Apr 1
EPI_ISL_447019	hCoV-19/Thailand/Bangkok-0078/2020	2020 Apr 1
EPI_ISL_496555	hCoV-19/India/MH-NCCS-NR048/2020	2020 Apr 28
EPI_ISL_480130	hCoV-19/Japan/PG-0470/2020	2020 Apr 1
EPI_ISL_462418	hCoV-19/Singapore/300/2020	2020 Apr 1
EPI_ISL_447359	hCoV-19/Israel/2089866/2020	2020 Apr 1
EPI_ISL_501210	hCoV-19/Malaysia/3998/2020	2020 Apr 1
EPI_ISL_447593	hCoV-19/Taiwan/TSGH-34/2020	2020 Apr 1
EPI_ISL_497818	hCoV-19/Hong Kong/HKU-200723-051/2020	2020 Apr 1
EPI_ISL_437192	hCoV-19/Indonesia/JK-EIJK-04/2020	2020 Apr 1

GISAID ID	Strain	Collection date*
EPI_ISL_420144	hCoV-19/Georgia/Tb-1679/2020	2020 Apr 1
EPI_ISL_491121	hCoV-19/Oman/8556/2020	2020 Apr 2
EPI_ISL_455707	hCoV-19/Vietnam/VNHN_4189/2020	2020 Apr 2
EPI_ISL_520729	hCoV-19/United Arab Emirates/L920638060/2020	2020 Apr 2
EPI_ISL_582033	hCoV-19/Iran/1600/2020	2020 Apr 3
EPI_ISL_483556	hCoV-19/Bahrein/BAH-15/2020	2020 Apr 4
EPI_ISL_450188	hCoV-19/Jordan/SR-0336/2020	2020 Apr 6
EPI_ISL_468077	hCoV-19/Bangladesh/CHRF-0009/2020	2020 Apr 8
EPI_ISL_456606	hCoV-19/Timor-Leste/TL07/2020	2020 Apr 11
EPI_ISL_454576	hCoV-19/Kazakhstan/16183/2020	2020 Apr 14
EPI_ISL_575330	hCoV-19/Beijing/NPRC0004/2020	2020 Apr 15
EPI_ISL_444969	hCoV-19/Guangdong/SYSU-IHV/2020	2020 Apr 16
EPI_ISL_525474	hCoV-19/Sri Lanka/CDR142/2020	2020 Apr 19
EPI_ISL_512844	hCoV-19/Myanmar/MMC_137/2020	2020 Apr 22
EPI_ISL_496526	hCoV-19/India/MH-NCCS-NR2257/2020	2020 May 21
EPI_ISL_491170	hCoV-19/Oman/C-11911/2020	2020 May 1
EPI_ISL_486910	hCoV-19/Japan/TK-Y17904/2020	2020 May
EPI_ISL_477169	hCoV-19/Georgia/Tb-7851/2020	2020 May 1
EPI_ISL_513176	hCoV-19/Saudi Arabia/KAUST-MADINAH684/2020	2020 May 1
EPI_ISL_528692	hCoV-19/United Arab Emirates/2/2020	2020 May 2
EPI_ISL_477128	hCoV-19/Bangladesh/CHRF-0015/2020	2020 May 2
EPI_ISL_510549	hCoV-19/South Korea/KCDC2211/2020	2020 May 3
EPI_ISL_462412	hCoV-19/Singapore/294/2020	2020 May 3
EPI_ISL_454585	hCoV-19/Kazakhstan/33496/2020	2020 May 4
EPI_ISL_501200	hCoV-19/Malaysia/3097/2020	2020 May 4
EPI_ISL_512991	hCoV-19/Saudi Arabia/KAUST-JEDDAH722/2020	2020 May 4
EPI_ISL_529966	hCoV-19/Indonesia/JI-ITD-7061V/2020	2020 May 5
EPI_ISL_498270	hCoV-19/Hong Kong/HKU-200723-105/2020	2020 May 8
EPI_ISL_548942	hCoV-19/Pakistan/UN-UVAS-Lahore-I/2020	2020 May 11
EPI_ISL_516884	hCoV-19/Israel/CVL-n17051/2020	2020 Jun 1
EPI_ISL_508202	hCoV-19/India/UP-AR66/2020	2020 Jun 9
EPI_ISL_514789	hCoV-19/South Korea/KCDC2386/2020	2020 Jun 1
EPI_ISL_477139	hCoV-19/Bangladesh/CHRF-0030/2020	2020 Jun 11
EPI_ISL_591352	hCoV-19/Japan/IC-0006/2020	2020 Jun
EPI_ISL_528694	hCoV-19/United Arab Emirates/306/2020	2020 Jun 2
EPI_ISL_482683	hCoV-19/Singapore/318/2020	2020 Jun 2
EPI_ISL_468159	hCoV-19/Pakistan/NIH-44905/2020	2020 Jun 2
EPI_ISL_582124	hCoV-19/Malaysia/MGI-MAEPS41/2020	2020 Jun 3

GISAID ID	Strain	Collection date*
EPI_ISL_513115	hCoV-19/Saudi Arabia/KAUST-MADINAH1111/2020	2020 Jun 3
EPI_ISL_574613	hCoV-19/Indonesia/JK-EIJK42/2020	2020 Jun 3
EPI_ISL_492014	hCoV-19/Oman/205041214/2020	2020 Jun 3
EPI_ISL_469254	hCoV-19/Beijing/IVDC-01-06/2020	2020 Jun 11
EPI_ISL_596455	hCoV-19/Iran/GRC-9695/2020	2020 Jun 11
EPI_ISL_525481	hCoV-19/Sri Lanka/CDR-SL7066/2020	2020 Jun 16
EPI_ISL_497770	hCoV-19/Hong Kong/HKU-200723-003/2020	2020 Jun 22
EPI_ISL_487270	hCoV-19/Bahrain/340217593/2020	2020 Jun 22
EPI_ISL_596558	hCoV-19/Palestine/84/2020	2020 Jun 22
EPI_ISL_582030	hCoV-19/Iraq/ICGEB-5T/2020	2020 Jun 30
EPI_ISL_498568	hCoV-19/Singapore/670/2020	2020 Jun 1
EPI_ISL_514266	hCoV-19/Israel/CVL-n25334/2020	2020 Jul
EPI_ISL_528707	hCoV-19/United Arab Emirates/581/2020	2020 Jul 1
EPI_ISL_522469	hCoV-19/South Korea/KCDC2689/2020	2020 Jul 1
EPI_ISL_497809	hCoV-19/Hong Kong/HKU-200723-042/2020	2020 Jul 1
EPI_ISL_596566	hCoV-19/Palestine/96/2020	2020 Jul
EPI_ISL_591539	hCoV-19/Japan/IC-0090/2020	2020 Jul
EPI_ISL_495023	hCoV-19/India/GJ-GBRC285/2020	2020 Jul 2
EPI_ISL_574433	hCoV-19/Indonesia/JK-NIHRD-C0027475/2020	2020 Jul 3
EPI_ISL_514246	hCoV-19/Bangladesh/BCSIR-NILMRC-381/2020	2020 Jul 21
EPI_ISL_497950	hCoV-19/Zhejiang/SX0715/2020	2020 Jul 15
EPI_ISL_525486	hCoV-19/Sri Lanka/CDR-KK57/2020	2020 Jul 21
EPI_ISL_498691	hCoV-19/Liaoning/IVDC-04/2020	2020 Jul 22
EPI_ISL_596449	hCoV-19/Malaysia/IMR-WI085/2020	2020 Jul 27
EPI_ISL_526745	hCoV-19/South Korea/KCDC2811/2020	2020 Aug 1
EPI_ISL_591460	hCoV-19/Japan/IC-0119/2020	2020 Aug
EPI_ISL_512842	hCoV-19/Singapore/806/2020	2020 Aug 2
EPI_ISL_528753	hCoV-19/Indonesia/JB-TFRIC19-R53817/2020	2020 Aug 4
EPI_ISL_561341	hCoV-19/India/HR-IMT-BZ164/2020	2020 Aug 25
EPI_ISL_512920	hCoV-19/Saudi Arabia/KAUST-JEDDAH462/2020	2020 Aug 4
EPI_ISL_536411	hCoV-19/Hong Kong/MV2007-00689/2020	2020 Aug 8
EPI_ISL_534336	hCoV-19/Taiwan/NTU30/2020	2020 Aug 11
EPI_ISL_596523	hCoV-19/Palestine/40/2020	2020 Aug 15
EPI_ISL_582642	hCoV-19/United Arab Emirates/skmc-3119719/2020	2020 Aug 25
EPI_ISL_575332	hCoV-19/Israel/CVL-s2049/2020	2020 Sep 1
EPI_ISL_576383	hCoV-19/Indonesia/JT-UGM-47906/2020	2020 Sep 1
EPI_ISL_577714	hCoV-19/India/KA-NIV-QC-1803/2020	2020 Sep
EPI_ISL_582659	hCoV-19/United Arab Emirates/skmc-3358771/2020	2020 Sep 3

GISAID ID	Strain	Collection date*
EPI_ISL_536429	hCoV-19/Singapore/888/2020	2020 Sep 3
EPI_ISL_418327	hCoV-19/Canada/ON-PHL-4181/2020	2020 Jan
EPI_ISL_404895	hCoV-19/USA/WA1/2020	2020 Jan 19
EPI_ISL_418344	hCoV-19/Canada/ON-PHL-2259/2020	2020 Feb
EPI_ISL_411954	hCoV-19/USA/CA-CDC-7/2020	2020 Feb 6
EPI_ISL_412972	hCoV-19/Mexico/CMX-InDRE-01/2020	2020 Feb 27
EPI_ISL_417139	hCoV-19/USA/WA-S86/2020	2020 Mar 1
EPI_ISL_418340	hCoV-19/Canada/ON-PHL-1083/2020	2020 Mar
EPI_ISL_509712	hCoV-19/Belize/CDC-6846/2020	2020 Mar 1
EPI_ISL_523811	hCoV-19/Dominican Republic/ICGEB_UNIBE051/2020	2020 Mar 1
EPI_ISL_424667	hCoV-19/Mexico/CMX-InDRE_03/2020	2020 Mar 4
EPI_ISL_415152	hCoV-19/Panama/328677/2020	2020 Mar 6
EPI_ISL_491438	hCoV-19/Costa Rica/INC-0026/2020	2020 Mar 6
EPI_ISL_450793	hCoV-19/Jamaica/JM-CDC-0869/2020	2020 Mar 9
EPI_ISL_509695	hCoV-19/Guatemala/CDC-1227/2020	2020 Mar 13
EPI_ISL_513312	hCoV-19/Cuba/USAFSAM-S030/2020	2020 Mar 19
EPI_ISL_434541	hCoV-19/Puerto Rico/CDC-S1/2020	2020 Mar 23
EPI_ISL_430130	hCoV-19/USA/WA-S276/2020	2020 Apr 1
EPI_ISL_467423	hCoV-19/Canada/BC_13704688/2020	2020 Apr
EPI_ISL_493335	hCoV-19/Mexico/GUA-InDRE-20/2020	2020 Apr 2
EPI_ISL_509714	hCoV-19/Belize/CDC-6852/2020	2020 Apr 9
EPI_ISL_462681	hCoV-19/USA/MI-MDHHS-SC20599/2020	2020 May 1
EPI_ISL_477039	hCoV-19/Canada/BC_00740314/2020	2020 May
EPI_ISL_525467	hCoV-19/Dominican Republic/ICGEB_UNIBE022/2020	2020 May 5
EPI_ISL_516622	hCoV-19/Mexico/SON-InDRE_35/2020	2020 May 15
EPI_ISL_477309	hCoV-19/USA/MN-MDH-1220/2020	2020 Jun 1
EPI_ISL_548900	hCoV-19/Canada/ON-PHL-20-01296/2020	2020 Jun
EPI_ISL_512659	hCoV-19/Costa Rica/INC-0053/2020	2020 Jun 8
EPI_ISL_516618	hCoV-19/Mexico/COA-InDRE-36/2020	2020 Jun 13
EPI_ISL_501083	hCoV-19/USA/OR-UW-13394/2020	2020 Jul 1
EPI_ISL_527750	hCoV-19/Costa Rica/INC-0082/2020	2020 Jul 2
EPI_ISL_582494	hCoV-19/Canada/MB-NML-1176/2020	2020 Jul 3
EPI_ISL_516611	hCoV-19/Mexico/BCN-InDRE-51/2020	2020 Jul 5
EPI_ISL_517928	hCoV-19/USA/FL-BPHL-1038/2020	2020 Aug 1
EPI_ISL_522880	hCoV-19/Mexico/CMX-INMEGEN-09/2020	2020 Aug 1
EPI_ISL_582478	hCoV-19/Canada/MB-NML-1152/2020	2020 Aug 1
EPI_ISL_536661	hCoV-19/USA/NY-UW-1289/2020	2020 Sep 1
EPI_ISL_408976	hCoV-19/Australia/NSW02/2020	2020 Jan 22

GISAID ID	Strain	Collection date*
EPI_ISL_410717	hCoV-19/Australia/QLD03/2020	2020 Feb 5
EPI_ISL_413490	hCoV-19/New Zealand/20VR0174/2020	2020 Feb 27
EPI_ISL_498500	hCoV-19/Australia/ACT0033/2020	2020 Mar
EPI_ISL_416519	hCoV-19/New Zealand/20VR0189/2020	2020 Mar 2
EPI_ISL_445000	hCoV-19/Guam/GU_NHG_03/2020	2020 Mar 20
EPI_ISL_498518	hCoV-19/Australia/ACT0051/2020	2020 Apr
EPI_ISL_579229	hCoV-19/New Zealand/20VR2101/2020	2020 Apr 1
EPI_ISL_456476	hCoV-19/Australia/VIC1511/2020	2020 May 1
EPI_ISL_579426	hCoV-19/New Zealand/20VR3128/2020	2020 May 1
EPI_ISL_498543	hCoV-19/Australia/ACT0084/2020	2020 Jun
EPI_ISL_548104	hCoV-19/New Zealand/20CV0063/2020	2020 Jun 19
EPI_ISL_498544	hCoV-19/Australia/ACT0086/2020	2020 Jul
EPI_ISL_548139	hCoV-19/New Zealand/20VR3804/2020	2020 Jul 10
EPI_ISL_563193	hCoV-19/Australia/VIC11004/2020	2020 Aug 1
EPI_ISL_547977	hCoV-19/New Zealand/20CV0068/2020	2020 Aug 12
EPI_ISL_563288	hCoV-19/Australia/VIC11186/2020	2020 Sep 1
EPI_ISL_548067	hCoV-19/New Zealand/20CV0220/2020	2020 Sep 1
EPI_ISL_412964	hCoV-19/Brazil/SP-01/2020	2020 Feb 25
EPI_ISL_414014	hCoV-19/Brazil/SP-03/2020	2020 Mar 2
EPI_ISL_414577	hCoV-19/Chile/Talca-1/2020	2020 Mar 2
EPI_ISL_482468	hCoV-19/Peru/LIM-01-2/2020	2020 Mar 5
EPI_ISL_418262	hCoV-19/Colombia/DC-INS-78390/2020	2020 Mar 6
EPI_ISL_420600	hCoV-19/Argentina/C121/2020	2020 Mar 7
EPI_ISL_547450	hCoV-19/Curacao/CW-RIVM-10092/2020	2020 Mar 10
EPI_ISL_547445	hCoV-19/Aruba/AW-RIVM-10101/2020	2020 Mar 13
EPI_ISL_444493	hCoV-19/Uruguay/Mdeo-1/2020	2020 Mar 13
EPI_ISL_477014	hCoV-19/Ecuador/USFQ-004/2020	2020 Mar 30
EPI_ISL_476373	hCoV-19/Brazil/SP-L22-CD474/2020	2020 Apr 1
EPI_ISL_498163	hCoV-19/Colombia/COR-INS-95319/2020	2020 Apr 1
EPI_ISL_536523	hCoV-19/Peru/LAM-INS-125/2020	2020 Apr 1
EPI_ISL_445349	hCoV-19/Chile/Santiago_52/2020	2020 Apr 1
EPI_ISL_430803	hCoV-19/Argentina/PAIS-A0012/2020	2020 Apr 1
EPI_ISL_476702	hCoV-19/Venezuela/VEN-89312/2020	2020 Apr 2
EPI_ISL_457965	hCoV-19/Uruguay/UY-NYUMC869/2020	2020 Apr 3
EPI_ISL_491933	hCoV-19/Ecuador/USFQ-128/2020	2020 Apr 7
EPI_ISL_468758	hCoV-19/Chile/Copiapo-00063/2020	2020 May 1
EPI_ISL_517613	hCoV-19/Suriname/SR-02/2020	2020 May
EPI_ISL_524786	hCoV-19/Brazil/PA-IEC-165302/2020	2020 May 1

GISAID ID	Strain	Collection date*
EPI_ISL_491941	hCoV-19/Ecuador/USFQ-133/2020	2020 May 2
EPI_ISL_536502	hCoV-19/Peru/CAL-INS-103/2020	2020 May 2
EPI_ISL_526933	hCoV-19/Colombia/AMA-INS-105161/2020	2020 May 3
EPI_ISL_476561	hCoV-19/Argentina/Heritas_HG006/2020	2020 May 7
EPI_ISL_491951	hCoV-19/Ecuador/52438/2020	2020 Jun 1
EPI_ISL_517620	hCoV-19/Suriname/SR-09/2020	2020 Jun
EPI_ISL_547571	hCoV-19/Brazil/SP-341/2020	2020 Jun 1
EPI_ISL_591528	hCoV-19/Chile/Santiago-ADC1858/2020	2020 Jun 8
EPI_ISL_529067	hCoV-19/Peru/LAM-UPCH-0006/2020	2020 Jun 8
EPI_ISL_547919	hCoV-19/Peru/UN-INS-003/2020	2020 Jul 1
EPI_ISL_486842	hCoV-19/Ecuador/USFQ-105/2020	2020 Jul 1
EPI_ISL_529139	hCoV-19/Brazil/RJ-DCVN2/2020	2020 Jul 20
EPI_ISL_517657	hCoV-19/Suriname/SR-46/2020	2020 Jul 7
EPI_ISL_526967	hCoV-19/Colombia/DC-INS-289/2020	2020 Jul 7
EPI_ISL_591527	hCoV-19/Chile/Santiago-LAE7918/2020	2020 Jul 28
EPI_ISL_527809	hCoV-19/Ecuador/USFQ-193/2020	2020 Aug 4
EPI_ISL_523959	hCoV-19/Brazil/SP-163/2020	2020 Aug 15
EPI_ISL_526971	hCoV-19/Colombia/DC-INS-197/2020	2020 Aug 18
EPI_ISL_591531	hCoV-19/Chile/Santiago-IN233883987/2020	2020 Aug 24
EPI_ISL_568518	hCoV-19/Peru/LIM-UPCH-0122/2020	2020 Aug 27
EPI_ISL_574431	hCoV-19/Ecuador/USFQ-253/2020	2020 Sep 14
EPI_ISL_417186	hCoV-19/South Africa/R03006/2020	2020 Mar 7

*Exact dates were not available for some sequences.

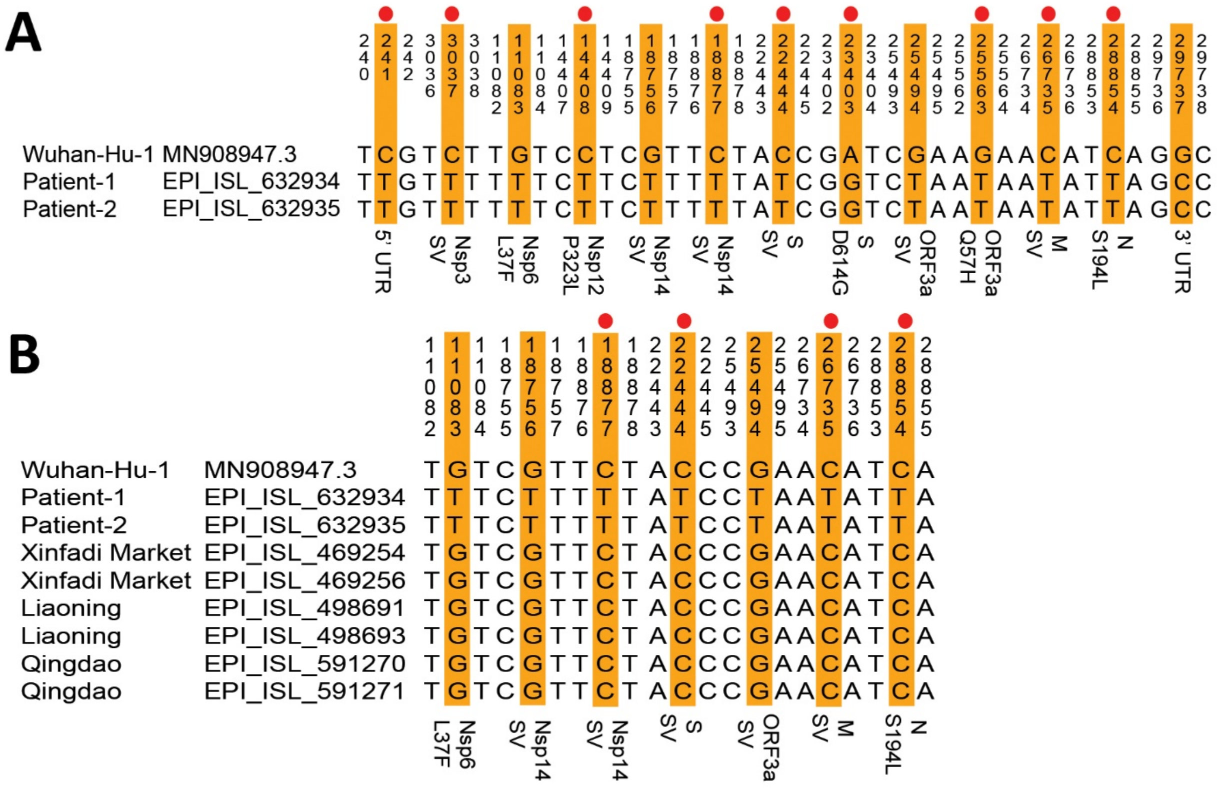

Appendix Figure 1. Reverse transcription PCR results of 2 undocumented migrants with severe acute respiratory syndrome coronavirus 2 (SARS-CoV-2), Yunnan Province, China, 2020. Cycle threshold (C_t) values for patient 1 (A) and patient 2 (B). Colored amplification curves indicate the results of the sequences: blue, ORF1ab gene; green, N-gene; and orange, internal control. The C_t value corresponds to the number of cycles at the intersection of the amplification curve and the threshold line. Patient 1 had no C_t value for the ORF1ab gene and a value of 37.41 for N-gene; patient 2 had C_t values of 31.23 for the ORF1ab gene and 30.11 for N-gene. NA, not available; RFU, relative fluorescence unit.

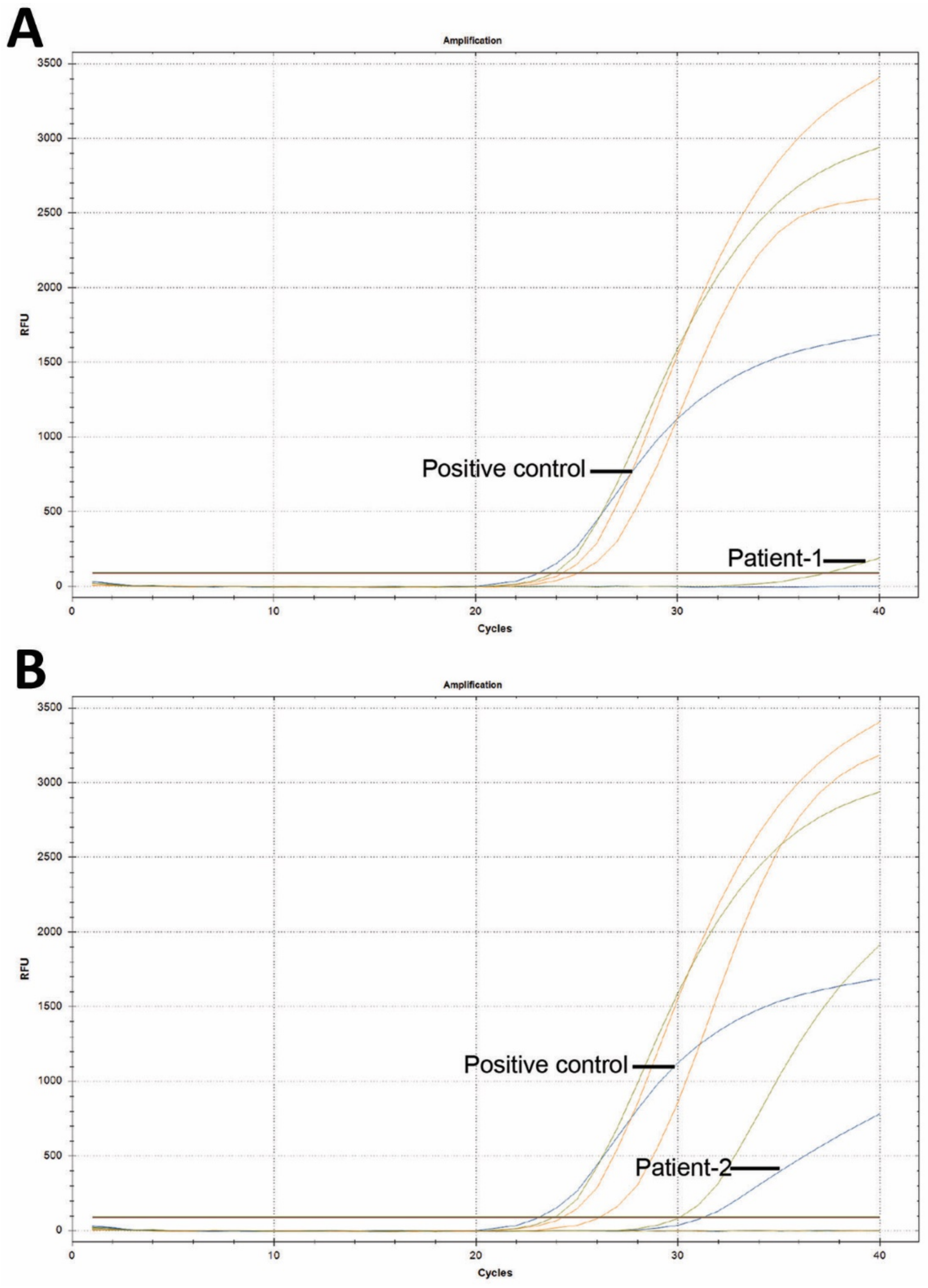

Appendix Figure 2. Comparison of severe acute respiratory syndrome coronavirus 2 sequences from earlier outbreaks in China and undocumented migrants, Yunnan Province, China, 2020. Wuhan-Hu-1 (GenBank accession no. MN908947.3) serves as reference sequence. Red circles indicate 9 representative mutations of B.1.36 clade.

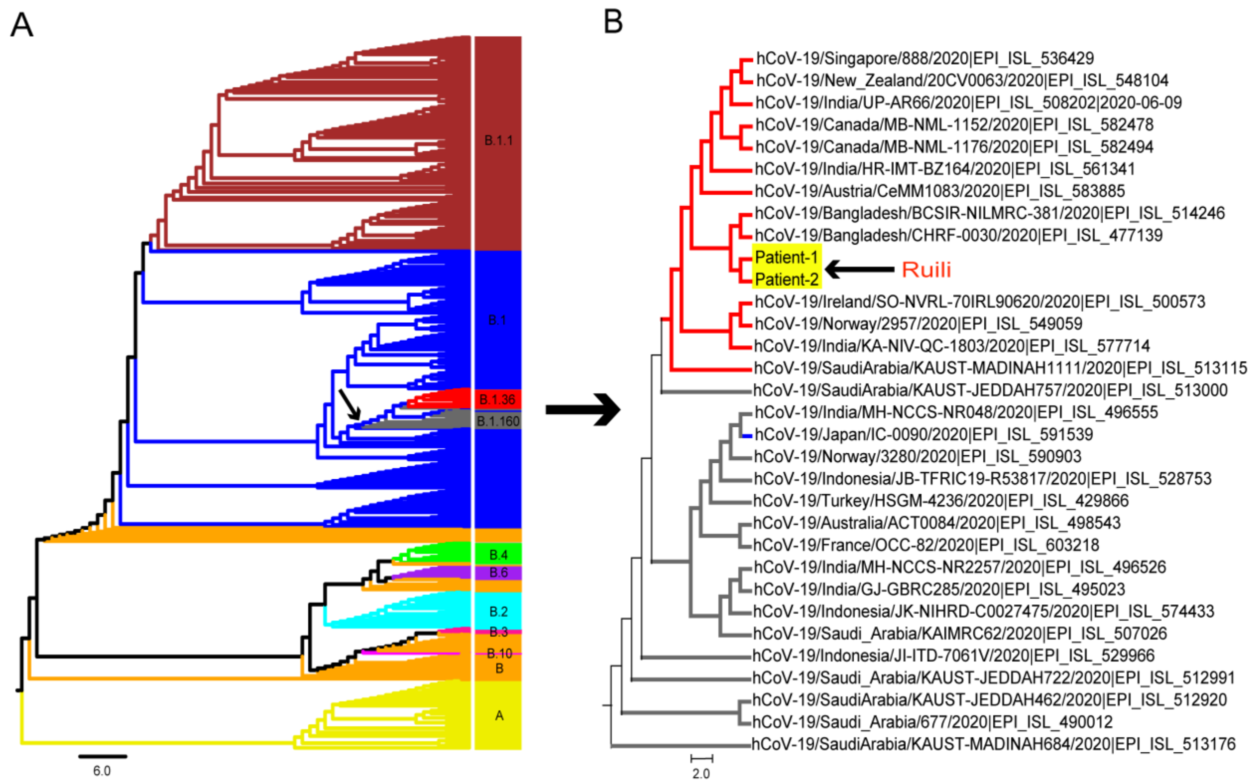

Appendix Figure 3. Global phylogenetic tree of severe acute respiratory syndrome, 2020. A) Phylogenetic tree of 592 sequences from GISAID (https://www.gisaid.org), including reference genome Wuhan-Hu-1 and 2 sequences from undocumented migrants in Ruili, Yunnan Province, China. Colors indicate different evolutionary lineages: yellow, A; orange, B; blue, B.1; brown, B.1.1; red, B.1.36; gray, B.1.160; cyan, B.2; pink, B.3; green, B.4; purple, B.6; and magenta, B.10. Black arrow indicates sequences from Ruili. B) Enlarged schematic of B.1.36 and B.1.160 lineages. Yellow bar indicates sequences from undocumented migrants in Ruili.
